# Respiratory diseases and allergy in farmers working with livestock: a EAACI position paper

**DOI:** 10.1186/s13601-020-00334-x

**Published:** 2020-07-06

**Authors:** T. Sigsgaard, I. Basinas, G. Doekes, F. de Blay, I. Folletti, D. Heederik, A. Lipinska-Ojrzanowska, D. Nowak, M. Olivieri, S. Quirce, M. Raulf, J. Sastre, V. Schlünssen, J. Walusiak-Skorupa, A. Siracusa

**Affiliations:** 1 Department of Environment Occupation & Health, Dept of Public Health, Danish Ramazzini Centre, Aarhus University, Bartholins Allé 2, Build. 1260, 8000 Aarhus C, Denmark; 2 Institute of Occupational Medicine, Edinburgh, UK; 3 Division of Environmental Epidemiology, Institute for Risk Assessment Sciences, Utrecht University, Utrecht, The Netherlands; 4 Division of Asthma and Allergy, Department of Chest Diseases, University Hospital, Fédération de Médecine Translationnelle de Strasbourg, Strasbourg University, Strasbourg, France; 5 Occupational Medicine, Terni Hospital, University of Perugia, Perugia, Italy; 6 Department of Occupational Diseases and Environmental Health, Nofer Institute of Occupational Medicine, Lodz, Poland; 7 Institute and Clinic for Occupational, Social and Environmental Medicine, University Hospital, Ludwig Maximilian University, Munich, Germany; 8 Comprehensive Pneumology Center Munich, Member DZL, German Centre for Lung Research, Munich, Germany; 9 Unit of Occupational Medicine, Department of Diagnostics and Public Health, University of Verona, Verona, Italy; 10 Department of Allergy, Hospital La Paz Institute for Health Research (IdiPAZ) and CIBER de Enfermedades Respiratorias (CIBERES), Madrid, Spain; 11 IPA Institute for Prevention and Occupational Medicine of the German Social Accident Insurance, Institute of the Ruhr-Universität Bochum, Bochum, Germany; 12 Department of Allergy, Fundación Jiménez Díaz, CIBER de Enfermedades Respiratorias (Ciberes), Madrid, Spain; 13 Formerly Department of Clinical and Experimental Medicine, University of Perugia, Perugia, Italy

**Keywords:** Agriculture, Asthma, Farm animals, Rhinitis, Work-related

## Abstract

Farmers constitute a large professional group worldwide. In developed countries farms tend to become larger, with a concentration of farm operations. Animal farming has been associated with negative respiratory effects such as work-related asthma and rhinitis. However, being born and raised or working on a farm reduces the risk of atopic asthma and rhinitis later in life. A risk of chronic bronchitis and bronchial obstruction/COPD has been reported in confinement buildings and livestock farmers. This position paper reviews the literature linking exposure information to intensive animal farming and the risk of work-related respiratory diseases and focuses on prevention. Animal farming is associated with exposure to organic dust containing allergens and microbial matter including alive microorganisms and viruses, endotoxins and other factors like irritant gases such as ammonia and disinfectants. These exposures have been identified as specific agents/risk factors of asthma, rhinitis, chronic bronchitis, COPD and reduced FEV_1_. Published studies on dust and endotoxin exposure in livestock farmers do not show a downward trend in exposure over the last 30 years, suggesting that the workforce in these industries is still overexposed and at risk of developing respiratory disease. In cases of occupational asthma and rhinitis, avoidance of further exposure to causal agents is recommended, but it may not be obtainable in agriculture, mainly due to socio-economic considerations. Hence, there is an urgent need for focus on farming exposure in order to protect farmers and others at work in these and related industries from developing respiratory diseases and allergy.

## Background

Although their numbers have declined considerably in most developed countries, farm owners and farm workers still constitute a large professional group [1]. The last decades showed a strong tendency towards specialization and concentration, leading to fewer but bigger farms. Farming practices are changing with large-scale enterprises gradually replacing smaller scale traditional family farms [2, 3].

Farm workers are exposed to airborne dust, microbial agents, and gases, particularly in livestock farming in closed confinement buildings. The increased risks of respiratory disease, including work-related (WR) asthma, rhinitis, and enhanced lung-function decline compatible with chronic obstructive pulmonary disease (COPD), have been well-recognized and summarized in the 80s and 90s [4], and confirmed in more recent reviews. Although general recommendations to lower exposure levels have been published, there is little evidence that these have been effectively implemented, and the risks of respiratory health problems in farmers may have remained high [5–8].

Given the ongoing changes in agricultural practice, it is worthwhile to assess their impact on respiratory health of farm workers. On the other hand, farm life has since the late 90s become widely known as protective against type I allergic sensitization and disease—particularly for children living on livestock farms, while protection seemingly also extends into adulthood [9–11]. The widespread recognition of this ‘anti-atopy protective’ effect might however also have led to underestimation or disregard of farm WR respiratory health risks.

An EAACI task force therefore produced a systematic update of evidence from the last two decades with regard to:

- prevalence and incidence of asthma/wheezing, rhinitis/rhinoconjunctivitis, atopic sensitization, bronchitis, and COPD in livestock farmers.
- clinical features, pathogenic mechanisms and diagnosis of farm work-related respiratory disease.
- the ‘anti-allergy protection paradox’: that living on a farm may protect against, while farm work would enhance the risk of asthma and rhinitis.
- exposure: levels and determinants, and protective measures to lower exposure.

Another major occupational risk of farm work-associated microbial and dust exposures is hypersensitivity pneumonitis (HP)—a potentially serious lung disease caused by high microbial exposures, strong humoral IgG sensitization against their—mainly fungal—allergens, and immune complex-mediated inflammation. Since HP has been extensively reviewed in another recent EAACI position paper [12], it is here just mentioned, but not further discussed.

Schenker et al. [4] have previously comprehensively reviewed the relevant published literature prior to the year 2000. For the present study extensive searches were therefore performed in literature from the last 18 years, with a primary focus on studies among farmers working with large animals/livestock (dairy and beef cattle, pigs, sheep, horses, poultry), and on respiratory symptoms and diseases and pulmonary function tests (wheezing, cough, asthma, rhinitis/rhinoconjunctivitis, chronic bronchitis, COPD and lower airway obstruction).

Results from three MEDLINE searches were combined (details in Appendix S1): 177 studies, 73 of which considered relevant to this document, were identified covering the years from 2000 through June 30, 2018. From the reference lists of relevant papers published since 2012 another 4 primary papers were added.

## Main text

### Epidemiology

Table 1 gives an overview of incidence and prevalence studies in livestock farmers, arranged by respiratory health outcome.

**Table 1:** Risk of asthma, rhinitis and respiratory symptoms and sensitization in farmers working with large animals: studies from 2000

References/country	Study design	Subjects (n)	Participation rate (%)	Age (years)	Animal exposures	Methods for defining rhinitis	Methods for defining asthma	Atopy assessment	Risk factors	WR asthma/rhinitis/respiratory symptoms: OR in farmers exposed to large animals (95% CI)
[8]/Denmark 2011 (SUS study)	Nested case–control study 4 years of FU	107 cases 102 controls		20^¤^	Swine dairy and chickens	Not done	SUS algorithm	SPT	Swine Dairy	New-onset asthma Exposure during FU: swine 3.4 (1.6–7.0) Dairy 2.5 (1.1–5.3) Corrected for Childhood exposure
[42]/Denmark 2018 (SUS study)	Follow up at age 35 for new onset sensiti-sation to common allergens; 15 years	1113 (of 1166)	50	20^¤^	Swine dairy and chickens	Not done		SPT and IgE	Endotoxin and dust Animal exposure	Endotoxin exp in quartiles associated to SPT: less sensitisation to cat allergens OR 0.1 → 0.6 and a tendency to increased loss of sen. to grass OR 3 → 4.2 IgE: less sensitisation to common allergens OR 0.4 → 0.8 and a tendency to increased loss of sense. Corrected for childhood exposure
[41]/Denmark 2018 (SUS study)	Follow up age 35 for new onset Lep D sensitisation 15 years FU	1116 (of 1166)	50	20^¤^	Swine dairy and chickens	Not done		SPT and IgE	Endotoxin and dust Animal exposure	Endotoxin exp in quartiles associated to SPT: more sensitisation OR 1.9 → 2.3 and decreased loss of sensitisation OR 0.1 → 0.2 IgE: more sensitisation OR 5 → 7 and decreased loss of sensitisation OR 0.1 → 0.7 Corrected for childhood exp
[56]/Germany, Denmark, Switzerland, Spain 2001	Cross-sectional	6156	61–80	48	Pig farmers	Q for nasal irritation	Q for wheezing	Not stated	Pig farmers only	Wheezing: pig farmers only 1.5 (1.2–2.0) Nasal irritation: pig farmers only 1.5 (1.2–1.9)
[15]/Turkey 2002	Cross-sectional	125	62	37	Grooms	Q	Q,	Not stated	Asthma: sensitization to horse hair Allergic rhinitis and conjunctivitis: being in the grooms group	Asthma: sensitization to horse hair 4.5 (1.5–13.3) Allergic rhinitis: groom 1.8 (1.0–3.1) Allergic conjunctivitis: groom 3.9 (1.6–6.6)
[141]/USA 2003	Cross-sectional	22,756	44	16–88	Beef cattle Dairy cattle Pig	Not reported	Q	Q	Wheeze: n. of animals on the farm, frequency of veterinary procedures, age, atopy Asthma: atopy	Wheeze: beef cattle 1.1 (0.98–1.1) Dairy cattle 1.3 (1.1–1.5) pig 1.1 (1.03–1.2) Any animal 1.1 (1.04–1.2)
[17]/Germany 2003	Cross-sectional	325	82	50	Sheep and other animals 37% Sheep shearing 24% Sheep dip 27% Chemical footbaths 66%	Q	Q	Q	Asthma-related sx: full time farming	Nasal allergy: 3.2 (2.1–4.6) Asthma-related sx 2.3 (1.2–4.3)
[16] USA 2009	Cross-sectional	82 72	80 34	41 38	Horse barns	Not done	Not Stated		Equine barn exposure 0; 1–10 and > 10 h/week Respiratory sx and nasal irritation: family history of respiratory problems and history of allergies	Respiratory sx: 2.3 (0.6–9.8) and 8.9 (3.3–32.3) in low and high exp Nasal irritation: 0.4 (0.6–1.5) & 3.5 (1.1–10.6) in low and high exposure In both analyses, family history of respiratory problems and history of allergies showed a significant association to increased symptoms OR of 5.3 and 8 for respiratory problems and 2.7 and 3.6 for Nasal irritation
[142]/USA 2017	Cross-sectional	11,210	71*	59.8	Crop 54% Livestock 46%	Not done	Rhinitis and Asthma D.D.	Not reported	Bale hay, Manure storage, grain, animals pesticides	Asthma and Rhinitis ass. to Pesticide spraying OR 1.9 (1.4–2.5) Rhinitis alone 1.3 (1.2–1.5) Ass to manure storage OR 0.71 (0.1–0.96)

*Q* Questionnaire, *WR* work-related, *sx* symptoms, *SPT* skin prick tests, *IgE* immunoglobulin E tests, *OR* odd ratios, *exp* exposure

* After exclusion of non-active farmers

^¤^At baseline

#### Asthma and wheeze

New onset asthma in farmers was reported in the Danish study of young farmers (SUS) [8], which found that during the first years after farming school the risk was significantly increased for work with swine [OR (95% cfi) = 3.4 (1.6–7.0)] and dairy cattle [OR = 2.5 (1.1–5.3)]. The risk was strongly associated with non-specific bronchial hyperresponsiveness (NSBHR) at baseline, but not with atopy, while a farm childhood was protective [OR = 0.5 (0.3–0.98)].

The European Community Respiratory Health Survey (ECRHS) follow up study found that new onset asthma was non-significantly associated with agricultural work in general [OR = 1.9 (0.7–5.2)], but did not discriminate between types of farm exposures [13].

In a range of other, cross-sectional studies, wheeze and asthma were associated with exposure to swine, dairy cattle, horse and sheep, but also with more specific exposures like manure (Table 1).

#### Rhinoconjunctivitis

Various cross-sectional studies have confirmed the previously well-established associations between nasal irritation and high dust exposures in farming. Increased ORs were reported for work with swine [OR = 1.5 (1.2–1.9) [14], work with horses and in horse stables [rhinitis OR = 1.8 (1.0–3.1)]; conjunctivitis [OD = 3.9 (1.6–6.6)] [15], for ‘highly exposed’ horse barn workers [OR = 3.5 (1.1–10.6)] [16] and in sheep breeders [OR = 3.2 (2.1–4.6)] [17].

Kronqvist et al. reported that rhino-conjunctivitis among farmers on the isle of Øland in Sweden was associated with dust mite sensitization, and that this sensitization was related to the time in farming, and thus work-related [18].

#### Chronic bronchitis and COPD

Chronic bronchitis (traditionally used to define COPD) has been statistically significantly associated with various dusty environments, including farms of different trades with point estimates for work with livestock of OR 1.9 [19, 20], dairy cattle 1.2 to 4.7 [21, 22]; swine 3.2 to 4.3 [19, 23] and horses 1.6 to 2.3 [24, 25]. Increased risks of COPD were reported for livestock farmers [OR = 1.4 (1.1–2.6)] [20]; non-smoking farmers working in confinement buildings [OR = 6.6 (1.1–40)] [26] and traditional farming [OR = 5.2 (1.7–16)] [27]. One study found associations with 3 different exposures (i) dairy cattle [OR = 1.8 (1.1–3)]; (ii) swine [2.3(1.1–4.9)] and (iii) poultry [2.6 (1.0–4.1)] [28] (Table 2). Thus, most animal husbandry is related to an increased prevalence of chronic bronchitis as well as COPD, with the highest relative risk in non-smoking farmers and female farm-workers from Concentrated Animal Feeding Operations (CAFOs) [23].

**Table 2:** Risk of chronic bronchitis, COPD and lung function decline in farmers working with large animals: studies from 2000

References/country	Study design	Subjects (n)	Participation rate (%)	Age (years)	Animal exposure	Methods for defining chronic bronchitis	Methods for defining bronchial obstruction	Risk factors	Chronic bronchitis OR in exposed to large animals (95% CI) unless otherwise stated	Bronchial obstruction/COPD OR in exposed to large animals (95% CI) unless otherwise stated
Iversen and Dahl [30] Denmark 2000	Longitudinal FU = 7 years	177	76	43 Baseline	Swine confinement and dairy farmers	Not done	Lung function^§^	Work exclusively with pigs or dairy	Not done	Swine confinement farmers: accelerated decline in FEV_1_ 53 mL year^−1^ vs. 36 mL year^−1^ in dairy non-smoking farmers, (p = 0.02)
Chaudemanche et al. [31]/France 2003	Longitudinal FU = 6 years	215	81	52 FU	Dairy farmers	Questionnaire	Lung function^§^	Chronic bronchitis and bronchial obstruction: dairy farming	Higher prevalence of chronic bronchitis in dairy farmers (7.5%) than in controls (1.8%, p < 0.02) PRR = 4.2	Decline in FEV_1_/VC ratio was significantly higher in dairy farmers than in controls -0.3 (SE 0.13) year^−1^ in a multiple linear regression correcting for smoking height, age, sex and altitude and initial value
Gainet et al. [32]/France 2007	Longitudinal FU = 12 years	157 farmers 159 controls	77 Calculated	51 FU	Dairy farmers		Lung function^§^			Farming Accelerated decline in FEV_1_/VC -1.2 ± 0.07% year^−1^ (p < 0.01) Corr. smoking height, age, sex and altitude
Thaon et al. [22]/France 2011	Longitudinal FU = 12 years	219 LF: 157	83	58 FU	Dairy farmers	Questionnaire	Lung function decline inFEV1/FV^§^	Usual morning phlegm: handling hay, straw and animal feed	Dairy farming: Morning phlegm: 4.3 (1.4–13) chronic bronchitis: 4.7 (0.5–41)	Dairy farming Accelerated decline in FEV_1_/FVC -0.21 ± 0.08% year^−1^ (p = 0.01) Animal feed: Accelerated decline in FEV_1_ 9.12 ± 4.7 ml year^−1^ (p = 0.05) Corr. For smoking height, age, sex and altitude
Bolund et al. [29]/Denmark 2015	Longitudinal FU = 15 yrs	1134	52	18.7 baseline	Farmers Swine and or dairy	Interview	lln^§^	Dairy, swine, LPS, Dust Farm upbringing	Not done	Current farming Accelerated decline in z-scores ∆FEV_1_ − 0.12 (− 0.2 to − 0.1) year^−1^ and ∆FEV_1_/FVC − 0.15 (− 0.3 to − 0.04) year^−1^. Corrected for smoking, second hand smoking, sex, being raised on a farm, baseline BHR and follow-up BMI Farm upbringing protective for decline in ∆FEV_1_ & ∆FEV_1_/FVC
Magarolas et al. [21]/Spain 2000	Cross-sectional	808	68	Not stated	Sheep workers Dairy farming	Questionnaire	Not done	Dairy farming	Chronic bronchitis: dairy farming 1.8 (1.1–2.9)	Not done
Kimbell-Dunn et al. [24]/New Zealand 2001	Cross-sectional	1706	78	Not stated	Beef/dairy cattle farmers 75%* Sheep 50%* Horses 15%	Postal questionnaire	Not done	Chronic bronchitis: horses, smoking, atopy	Chronic bronchitis: working with horses 1.6 1.1–2.5)	Not done
Radon and Winter [17]/Germany 2003	Cross-sectional	325	82	50	Sheep and other animals 37% Sheep shearing 24% Use of sheep dip 27% Use of chemical footbaths 66%	Questionnaire	Not done	Chronic bronchitis: sheep breeding ODTS: sheep breeding & footbaths	Chronic bronchitis: full time farmers 1.9 (0.9–3.9)	Not done
Monsò et al. [26]/Europe 2004	Cross-sectional	105 non-smokers	85	45	Confinement buildings: Pig farmers 78%* Beef/veal f. 30%* Dairy f. 22%* Poultry f. 31%*	Questionnaire	Lung function	COPD: organic dust (dose–response relationship)	Not reported	COPD in non-smoking farmers working inside confinement buildings: organic dust 6.6 (1.1–39.5)
Schenker et al. [19]/USA 2005	Cross- sectional	1947 1751 m 196 f	80 by contact 43 by target pop	54 m 54f	Livestock 13%	Questionnaire	Not done		Chronic bronchitis prevalence: female swine farmers 3.9% Asthma related to livestock last 12 months 12%	
Senthilselvan et al. [23]/Canada 2007	Cross-sectional	374	70	36	Full time swine farmers	Questionnaire	Lung function	Chronic bronchitis: full time swine farming and female sex	Chronic bronchitis: Female sw farmers 4.3 (1.9–9.7) Male sw farmers 3.2 (1.8–5.9)	No differences in lung function among swine farmers and controls and among females and males
Gallagher et al. [25]/NZ 2007	Cross-sectional	475 318	72 64	53.3 49.4	475 horse trainers 318 vegetable growers	Questionnaire	Not done	Chronic bronchitis	Chronic bronchitis prev. 8 vs 3% OR for CB increased in horse tr 2.3 (1.1–5.2) c f age, gender, smoking, family history of atopic conditions, and dust exposures outside of work	
Eduard et al. [20]/Norway 2009	Cross-sectional	4469	90	15–29 years 1496 30–49 years 1647 50–70 years 1326	Livestock farmers	Questionnaire	Lung function	Livestock farming, ammonia, hydrogen sulfide dust and atopy	Chronic bronchitis: 1.9 (1.4–2.6)	COPD: 1.4 (1.1–2.6) FEV_1_ was significantly reduced
Elfman et al. [143]/2009 Sweden 2009	CS Tox	13	??		Horse grooms visited 3 times spring–summer spring 2004–2005	Questionnaire NAL	Too small to see effects			
Tual et al. [144]/France 2013	Cross-sectional	14,441	99	65	Cattle farmers 68%* Poultry f. 30%* Pigs f. 24%*	Questionnaire	Not done	Cattle raising Small-scale cattle raising	Chronic bronchitis: cattle farmers 1.2 (1.03–1.5) Non-smoking cattle farmers 1.5 (1.1–5.9)	Not done
Viegas et al. [145]/Poland 2013	Cross-sectional	33 70			Swine barn workers Persons with no ag work	Interviews		Swine barns	Asthma n = 3 (12%) Wheezing n = 10 (35%) Coughing n = 12 (41%) Dose response for symptoms	Not done
Rodriquez et al. [146]/USA 2014	Cross-sectional	450 Hispanics	na	22–70	Hired farm workers	Interview	Lung function	Farming	Dairy farming	
Mitchell et al. [147]/USA 2015	Cross-sectional		205 45	91 92	Parlor workers Processing plant (pepper) Workers	Questionnaire	Lung function			Years worked in agr associated with ↓ FEV1*/*FEV6
Guillien et al. [28]/France 2016	Cross sectional	3787	41	40–75	Cattle breeders Swine breeders Poultry breeders Breeders of 2 + livestock types	Questionnaire	Lung function	Animal farming Geographical area	Not reported	COPD: cattle 1.8 (1.1–3.0) Swine 2.3 (1.1–4.9) Poultry 2.6 (1.0–4.1)
Marescaux et al. [27]/France 2016	Cross sectional	590	72	COPD lln − 53.9 + 59.0	Dairy farmers Doubs region	Questionnaire	Lung function	Farm size and modernity Smoking (sm)	Not reported	COPD lln Traditional Farm 5.20 (1.73–15.6) Interaction analysis Non-sm/modern 1 Sm/modern 1.33 (0.2–10) Non-sm/trad 5.39 (1.2–25) Sm/trad 8.29 (1.9–37)
Nonnenmann et al. [148]/USA 2017	Cross sectional	62	na	32 [10]	Milking cows	Interview	Not done			

*Lln* lower limit of normal, *na* not available, *COPD* chronic obstructive pulmonary disease, *OR* odd ratio, *PRR* proportional reporting ratio, *FU* follow up period, *LPS* Lipopolysaccharides

* Not mutually exclusive

^§^No post dilatation lung function performed

#### Lung function

The few follow up studies on lung function development clearly indicate an increased risk of obstructive changes over time (Table 2). However, the effects are modest according to a recent review [29]. Non-smoking Danish farmers showed an accelerated loss of forced expiratory flow in the first second (FEV_1_) of 53 ml per year among swine-breeders compared to 36 ml per year among dairy farmers [30]. Studies in France where the study population comprising of dairy farmers was followed for periods of 6 [31] and 12 years [32] showed an accelerated decline in Tiffeneau index (FEV_1_/VC) of 0.3 and 1.2% year^−1^ in comparison to controls. In a reinvestigation of the French 12 yr follow-up data an accelerated decline in FEV_1_/FVC was calculated of − 0.21 ± 0.08% year^−1^ among the dairy farmers and an accelerated decline in FEV_1_ of − 9.12 ± 4.7 ml year^−1^ in the group handling animal feed [22].

One study additionally reported a significant interaction for COPD between traditional farming and smoking with ORs of 5.4 for traditional farm, 1.3 for smoking and 8.3 for the combination of smoking and working on a traditional farm [27].

At 15 year follow-up in the Danish SUS study, a farm work-associated accelerated decline was noted for z-scores FEV_1_ (0.12 year^−1^) and FEV_1_/FVC (0.15 year^−1^). Furthermore NSBHR at baseline appeared to be a risk factor for decline in FEV_1_, but only in farmers without farm childhood. Interestingly, being raised on a farm was protective against a decline in FEV_1_ and FEV_1_/FVC during follow up [29].

Two cross-sectional studies have reported lung function in farmers with diverging results (Table 2). A smaller Canadian study in 375 swine farmers showed no differences in lung function between swine farmers and controls [33], whereas a greater more general study of 4735 Norwegian farmers found FEV_1_ significantly reduced among animal breeders compared to crop farmers [20].

In summary, the risk of obstructive lung function changes has remained high in farmers engaged with animals and animal feeding operations, or as an interaction between smoking and farm work exposures. However, the acceleration in lung function decline seems to be modest [34].

### Pathogenesis, clinical features, diagnosis, and protective effects

#### Pathogenic mechanisms

Asthma and rhinitis in farmers may vary from IgE-mediated allergy to specific farm allergens, to non-IgE-dependent innate immunity responses to microbial agents, or dust-, chemical-, or other irritant-induced airway reactivity [35].

Most reported specific type I allergies are to storage mite [20] and bovine allergens [39–42, 54], while IgE sensitization to horse allergens has been recognized as a growing problem in horse riders and horse stable workers [15, 36]. IgE to storage mites can be found in dairy farmers, and dust from their homes shows enhanced concentrations of storage mite allergens, e.g. *A. siro*, *L. destructor* and *T. putrescentiae* [37]; relations with storage mite sensitization and ensuing rhinitis and asthma are however not well-established. Dairy farmers are also exposed to bovine allergens and *Bos d2* is an important major allergen in cattle barns, also found in farm house dust [38–40].

However, there is a lack of population data to assess whether these high exposures to farm allergens are associated to WR rhinitis and asthma. Given the high exposure levels, the sensitization frequency among farmers is remarkably low—possibly as a result of the ‘anti-atopy’ protective effect of the farm environment, as discussed below. Interestingly, in the Danish follow-up study, new sensitization to storage mite (*Lep d*) was positively associated with farm work, whereas sensitization to common allergens tended to decrease at higher farm exposures [40–42].

**Fig. 1:**
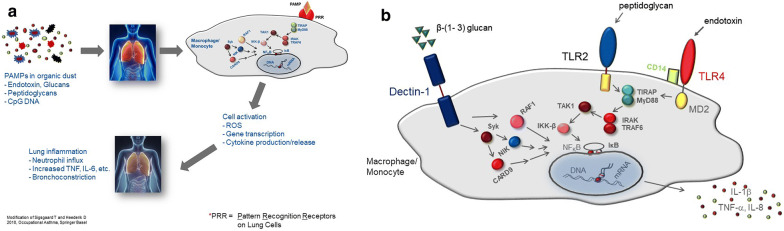
Mechanism PAMP-induced innate immunity responses to microbial agents. Examples for PAMPs (activators of the innate immune system) Endotoxin (LPS) signaling through TLR4-pathway expression TLR4 LPS induced inflammatory response (e.g. RSV increased TLR4) although LPS causes inflammation in everyone, people with asthma tend to be more sensitive several proteins are involved in LPS-response. Peptidoglycan signaling by TLR2 and, PGRPs (peptidoglycan recognition proteins), and NODs (nucleotide-binding oligomerization domain molecules) b(1 → 3)-glucans (polymers of glucose produced in fungi, plants and some bacteria) Dectin-1, expressed on macrophages and neutrophils, is the b-glucan receptor Dectin-1 may function as a T cell co-stimulatory molecule, suggesting that b-glucan stimulation may be a link between innate and adaptive immune response

Most work-related upper (URT) and lower respiratory tract (LRT) symptoms in farmers, however, are probably caused by non-IgE mediated, innate immunity responses to airborne agents of microbial origin, which are inhaled at high levels in livestock farming [43]. Many of the components of the bio-aerosols in stables are pathogen- or microbial-associated molecular patterns (PAMPs/MAMPs) that bind to specific receptor molecules and activate innate immunity pathways [44]. Inhaled PAMPs from bio-aerosols induce airway inflammation in healthy and asthmatic subjects and symptom exacerbations to a variable degree, likely depending on the burden of exposure and some polymorphisms in the endotoxin cell receptors and signal transduction molecules [44]. Airway inflammation starts in the case of endotoxin through the TLR4-pathway, peptidoglycan by TLR2-associated peptidoglycan recognition proteins (PGRPs), nucleotide-binding oligomerization domain (NODs) molecules and β(1 → 3)-glucans (polymers of glucose produced in fungi, plants and some bacteria) may act through the β-glucan receptor, Dectin-1, expressed on macrophages and neutrophils (Fig. 1).

Most intensively studied are the pathogenic mechanisms of wheezing and asthma in pig farming, especially in swine confinement buildings, where high and chronic airborne PAMP exposures may not only lead to local airway and lung inflammation, but also to systemic effects as shown by increased levels of circulating serum cytokines TNF-α, Il-6 and Il-1β [30, 45, 46] (Fig. 1). Symptoms are wheeze, coughing and other typical asthmatic symptoms and features like increased NSBHR [47–50]. In naïve subjects high exposures during a few hours in a pig stable may even lead to symptomatic systemic inflammation with increased body temperature, chills and malaise [48, 49]. Interestingly repeated Organic Dust Toxic Syndrome (ODTS) is associated with a fivefold increase in chronic phlegm risk [51].

### Clinical features

Farm work-related URT and LRT symptoms as such do not show typical features with which they might be distinguished from non-occupational cases. Asthma may have several phenotypes, such as IgE-mediated asthma characterized by high reversibility in airway obstruction [52] and non-atopic asthma with low reversibility, NSBHR and wheezing [35, 53]. Nasal symptoms such as congestion, rhinorrhea and pruritus are common in farm workers across the different areas in agriculture [4, 54] including veterinarians [55]. Several cross-sectional studies report nasal irritation without mentioning other symptoms of rhinitis while others described rhinitis combined with conjunctivitis. Among 6156 randomly selected animal farmers in Denmark, Germany, Switzerland and Spain, the prevalence of nasal irritation was 22% for farmers working with cattle, 29% for pig farmers, 21% for working with sheep and 22% for mixed farming [56].

The role of atopy-defined as positive skin prick or IgE tests to common allergens—is not always clear. In cases with specific type I allergy to farm allergens like storage mites or bovine allergens, sensitization to common allergens is one known risk factor [57, 58]. However, in a community based sample of farmers, no association was found between sensitization to cow dander and occupational symptoms [59]. In several studies in farmers and other agricultural workers the prevalence of common atopy was low (10–15%) compared to contemporary population studies (> 25%), but atopics were at higher risk to develop URT- and LRT-symptoms, including non-IgE mediated airway inflammation induced by microbial agents [10]. In contrast, in Danish young farmers prevalence and incidence of asthmatic disease was independent of common atopy, while NSBHR at baseline was a risk predictor [8].

Repetitive farming exposure can result in chronic lung inflammatory disease with significant decline in lung function over time [29, 30, 32]. In a substantial fraction of workers there might also be a “chronic inflammatory adaptation response” as a significant attenuation of the initial, robust inflammatory response following repetitive exposure, of which the precise mechanism is not clear [60]. Such tolerance is however definitely not a general feature common to all farm workers exposed to high levels of microbial dusts [10].

### Diagnosis

Diagnosis is complicated by the variety of etiologic agents and pathogenic mechanisms present in farming environments. Since the majority of cases may not be due to specific allergic sensitization to occupational allergens, negative results of skin prick or IgE tests may easily lead to a failure to identify farm-related causal factors. It is of crucial importance that the diagnostic anamnesis of a farm worker presenting with respiratory symptoms includes a careful inventory of work-related exposures that might induce or aggravate allergic symptoms. Practitioners must be well aware that neither atopic sensitization to common allergens, nor a lack of specific sensitization to farm allergens should be interpreted as negative evidence against farm exposures as primary or secondary causes of the farmer’s respiratory ailment. Asthma diagnosis is performed according to the statement by an earlier position paper [61]. In the presence of work-related rhinitis or asthma, serial recordings of nasal symptoms and peak flow measurements can be performed. In some cases objective assessment using provocation challenges in the laboratory or at the workplace can be recommended for asthma and rhinitis [62].

Diagnostic tests for specific allergies are only helpful in the minority of patients with type I allergies to farm-related antigens, e.g. in Finland where cow dander has been recognized as an important type I occupational allergen, since the majority of farmers with allergic rhinitis had a positive reaction to nasal challenge with cow dander [57]. Similarly, suspected type I allergy to storage mites or horse allergens may be tested with appropriate skin prick tests (SPTs) or IgE tests if available, but even in case of proven sensitization the link between exposure to the allergen and occurrence of symptoms must be confirmed by a careful anamnesis or by specific inhalation challenge (SIC) tests.

SICs with specific allergens can be conducted either with the suspected specific agent in the laboratory or at the subject’s workplace [61]. These tests should be conducted only by specialized centers. SICs may be especially useful when a) alternative procedures have failed to identify with sufficient accuracy the diagnosis of occupational allergy; b) the patient is no longer exposed at work; or c) there is need to identify a particular agent/s; d) if an agent has not previously been recognized as a causal factor; and e) for medico-legal requirements.

There is no single diagnostic test available to confirm or exclude a diagnosis of disease caused by innate immunity reactions to airborne PAMPs at the workplace. A controlled inhalation challenge test may be performed at the workplace, but the nature of innate immunity reactions implies that also naïve subjects may vigorously respond to such exposures. Hence, such challenges alone do not confirm a specific responsiveness to work-related exposure.

Nasal provocation tests can be performed also either in the laboratory under controlled conditions or at work under natural conditions to confirm the presence of occupational allergic rhinitis.

Nonspecific inhalation challenges—with e.g. histamine, methacholine, cold air or hypertonic saline—may be helpful in the diagnosis of asthma, as a positive reaction is a serious predictor of later onset asthma in young farmers [8]. In young farmers without a farm childhood, and thus relatively naïve to the farm environment, NSBHR was found to be associated with an increased decline in lung function over a 14 year follow up [29].

In general, the diagnosis of farm-related LRT and URT illness must primarily rely on a strong systematic anamnesis focusing on specific work tasks with high exposure. In some specific cases, such as in clusters of workforces with a sudden very high incidence of work-related symptoms, anamnesis should be supported by exposure measurements at the workplace, and monitoring of time and place when and where symptoms occur. Another issue to consider is, that endotoxin induced inflammation and NSBHR usually develop with a sub-acute pattern, i.e. not simultaneously with exposure, but most often start 4–8 h after exposure.

### Protection by the farm environment

Chronic exposure to animal farm dusts may also attenuate inflammatory responses and even protect against type I allergies. Adaptation to high endotoxin exposure has been described already > 30–40 years ago in cotton workers who showed the most vigorous responses after the weekend (hence called ‘monday morning fever’) or after a few weeks off-work, while after some days of exposure the acute inflammatory responses and symptoms became less severe [63, 64]. Similar effects have been found in experimental studies in which airway and systemic inflammation (measured as cytokines in nasal fluid and/or induced sputum, and in serum) and changes in NSBHR were compared between swine workers and healthy volunteers after exposure to swine barn dust [47, 65, 66]. Swine farmers had higher baseline levels of inflammatory markers, suggesting chronic airway inflammation, but responded less to acute exposures than naïve volunteers [47, 67]. The mechanisms behind this apparent “adaptation” to high airborne organic dust exposures are not known [68, 69], but probably similar to those of the much better studied ‘endotoxin tolerance’ of innate immunity cells in studies of endotoxin exposure due to life-threatening systemic bacterial infection [70–72]. If such mechanisms indeed also are operative in farmers with chronic microbial exposures, it would explain why adverse health effects in some studies may appear to be less severe than expected based on their high exposure levels. Healthy worker selection (HWS) may also be involved [33, 73, 74], but its role may vary among populations in different countries and types of farming [75].

However, it would be a serious misunderstanding to conclude that farm workers after some time become tolerant. Although acute responses may be attenuated, there is overwhelming evidence of ongoing chronic airway inflammation and a more rapid decline of lung function in populations highly exposed to PAMPs [44].

The other ‘beneficial’ effect of exposure to the livestock farm environment is the lower risk of allergic (atopic) asthma and rhinitis among those born and raised on a farm. These findings, published for young farmers [76], school children in Alpine regions [77–80] and confirmed in studies from many other countries [7, 10, 11, 42, 81–94], revived nineteenth century knowledge that hay fever is rare in farmers [95]. A commonly accepted explanation holds that the developing immune system of farm children is primed towards a state of non-atopic responsiveness or immune tolerance for allergens [42, 77, 81, 84, 96], by chronic inhalation of farm dust containing pro-inflammatory “microbe—associated molecular patterns” (MAMPs) (see paragraph on mechanisms), and/or by frequent ingestion of unpasteurized milk that also may contain enhanced concentrations of such MAMPs and in addition other agents with immunoregulatory properties like prebiotics and various cytokines; according to these theories it would be the very early or even prenatal farm exposures that protect against type I allergies. These protective effects might be most pronounced for traditional small-scale farming, as in children studied in the original reports from Alpine regions [77, 78, 80]. Other evidence for such an association restricted to more ‘old-fashioned’ farming comes from the study by Stein et al. [90] in the USA, who compared atopy in children from Amish communities who adhere to strict traditional farm practices, with children from the more modern Hutterite families. Lower risks of type I sensitization and type I allergic disease have however also been found in several other populations of both children and adults who grew up in the last decades in relatively modern farms, as in The Netherlands, Sweden [97–101] and Denmark [11, 42, 88].

Since many farm workers also have been raised on a farm, it is hard to assess these effects separately. Table 3 summarizes studies on the prevalence of atopy and atopic disease in farmers and non-farmers, with farm childhood also taken into account. In many studies, a farm childhood appeared to confer a long-lasting protection into adulthood [7, 10, 11, 82, 83, 85–89, 91–93, 101–103], while some also reported evidence that current farm work may additionally protect against sensitization to common allergens and/or atopic illness [88, 89, 96, 102, 103]. One longitudinal study found a lower risk of new pollen sensitization in young adulthood, especially in those with high animal stable dust and endotoxin exposures [42]. HWS bias seemed unlikely, since the frequencies of NSBHR and wheezing are higher or similar among the highly exposed workers, and protection in adulthood appeared to be mainly restricted to atopic sensitization. It especially pertained to hay fever, pollen sensitization [11, 42, 96, 101] and atopic asthma, while non-atopic wheezing and NSBHR are more prevalent at high farm dust exposures [8, 76, 89, 101, 104]. Thus, farm work-associated exposures may, in addition to a farm childhood, protect against persistence of, or newly originating atopic sensitization to pollen and possibly other common allergens [10, 11, 101].

**Table 3:** Effects of farm childhood and adult farm work/exposure on the risk of asthma/rhinitis/allergic sensitization in adulthood: Studies from 2000

References/country	Populations (n)/design	Farm childhood	Adult exposure	Asthma	Rhinitis	SPT	Specific IgE	Total IgE	Remarks
				OR (95% CI) unless otherwise stated					
Lampi et al. [92]/Finland 2011	Prospective birth cohort study; atopy at age 31	1262+ vs 4247−	Not done	Dd asthma ever: 0.7 (0.5–1.0)	Allergic rhinitis at age 31: 0.9 (0.7–1.03)	Positive SPT: 0.7 (0.6–0.8)	Not done	Not done	/
Omland et al. [8]/Denmark 2011 (SUS study)	Nested case–control study (107 vs 102)	77+ vs 132	Swine farming (n = 94) Dairy farming (n = 59)	New-onset asthma Farm childhood: 0.50 (0.2–0.98) Exposure during FU: Swine 3.4 (1.6–7.0) Dairy 2.5 (1.1–5.3)	Not done	Atopy (positive SPT): not a risk factor for new-onset asthma	Not done	Not done	/
Varraso et al. [149]/France 2012	54,018 female adults/13 years. follow-up	Farmer parents Place of birth Bovine density score 0–3	Not done	Farmer parents: childhood asthma 0.5 (0.4–0.7) adult-onset asthma 0.7 (0.6–0.8) Rural birth: childhood asthma 0.8 (0.7–0.9) adult-onset asthma 0.9 (0.8–0.96) Highest vs. lowest bovine density score: childhood asthma 0.7 (0.5–0.98) adult-onset asthma 0.8 (0.6–0.98)	Not done	Not done	Not done	Not done	Focus on asthma history and phenotype and on dietary factors Effects on both persistent and adult-onset asthma
Elholm et al. [11]/Denmark 2013 (SUS study)	1166/follow-up at age 35 for new sensitization to common allergens	496+ vs 476− (of 1162)	Farm work during follow-up (age 20–35)	Not stated	Not stated	No-farm childhood 0.6 (0.3–1.3) Farm childhood 0.4 (0.1–1.2)	No-farm childhood 0.2 (0.05–0.7) Farm childhood: too few subjects	Not done	/
Elholm et al. [42] Denmark 2018 (SUS study)	884 (of 1166) follow-up at age 35 for new onset sensitization					Farm Childhood OR = 0.5 Sensitisation to pollen during follow up vs sensitization in no-farm child	Farm Childhood OR = 0.5 Sensitisation to pollen during follow up vs sensitization in no-farm child	Not done	
Elholm et al. Denmark 2018 (SUS study)	1116 (of 1166)/follow-up at age 35 for new sensitization to Lep D	558 558	Farm work during follow-up (age 20–35)	Not done	Not done	Farm Childhood OR = 0.5 Sensitisation to endotoxin during follow up ass to less sensitization in no-farm child	Farm Childhood OR = 0.5 Sensitisation to endotoxin during follow up ass to less sensitization in no-farm child	Not done	
Kilpeläinen et al. [85]/Finland 2000	10,667 1st year university students/cross-sectional	1095+ vs 1243-	Not done	Farm childhood: dd asthma 0.7 (0.5–0.9)	Farm childhood: dd rhinitis 0.6 (0.5–0.8)	Not done	Not done	Not done	/
Ernst &Cormier [86]/Canada 2000	1199 secondary school children from rural areas, age 12–19 years/cross-sectional	802+ vs 397−	Not done	Farm childhood: wheeze 0.7 (0.6–0.99) dd asthma 0.7 (0.4–0.98)	Not done	Farm childhood: 0.6 (0.5–0.8)	Not done	Not done	Farm childhood: BHR 0.8 (0.6–0.9)
Leynaert et al. [87]/4 EU and NZ (ECRHS) 2001	6251 subjects 20–44 years of age/cross-sectional study in the general population	548+ vs 5703−	Not done	Farm childhood: current asthma 0.8 (0.5–1.39) wheeze 1.1 (0.8–1.5)	Farm childhood: pollen-related nasal sx 0.8 (0.6–1.02) animal/feather/dust-related sx 0.97 (0.8–1.2)	Not done	Farm childhood: 0.8 (0.6–0.97) cat sensitization 0.6 (0.4–0.96) grass s. 0.7 (0.5–0.9) hdm s. 0.8 (0.6–1.1) *Cladosporium* s. 0.9 (0.4–1.9)	Not stated	Between-country heterogeneity
Portengen et al. [88]/Denmark 2002	999 farming students age 19 years/cross-sectional	505+ vs 494−	Farming vs non-farming	Farm childhood: asthma 0.8 (0.5–1.3) wheeze 0.7 (0.4–1.1) Farmers: wheeze less often than controls (p < 0.05)	Farm childhood: rhino-conjunctivitis 0.7 (0.5–0.99)	Farm childhood: 0.5 (0.4–0.8) Farmers: + SPT lower than controls (p < 0.05)	Not stated	Farm childhood: 0.7 (0.5–1.1)	Farm childhood: BHR 0.6 (0.4–0.95)
Eduard et al. [104]/Norway 2004	1614 farmers/cross-sectional	Not done	JEM, farmers with livestock vs farmers without livestock	Asthma: cattle farmers 1.8 (1.1–2.8) pig farmers: 1.6 (1.0–2.5) Non-atopic asthma: pig farmers 2.0 (1.2–3.3) 2 + livestock 1.9 (1.1–3.3) Atopic asthma: 2 + livestock 0.3 (0.1–0.97)	Not done	Not done	Not stated	Not done	Atopic vs. non-atopic asthma
Radon et al. [102]/Germany 2004	3112 rural subjects, age 18–44 years/cross-sectional	1268+ vs 1807−	Presently living on farm	Presently living on a farm: atopic asthma sx 0.7 (0.4–1.4) non-atopic asthma 0.9 (0.6–1.4) Regular visits to stables started at age 4–6: atopic asthma sx 0.4 (0.2–0.95)	Presently living on a farm: nasal allergies 0.6 (0.4–0.9) Regular visits to stables started at age 4–6: nasal allergies 0.4 (0.2–0.6)	Not done	Not done	Not done	/
Koskela et al. [150]/Finland *2005*	231 women living on a farm, 202 women not living on a farm/cross-sectional	119+ vs 314−	Presently living on farm	Not done	Not done	+ SPT: living in a dairy farm 35%, not living on a dairy farm 37% (NS) Sensitization to pollens: living in a dairy farm 4.4%, not living on a dairy farm 17.3% (p = 0.01) S. to cat: living in a dairy farm 3.5%, not living on a dairy farm 10.4% (p < 0.05)	Not done	Not done	Protection by living on a dairy farm only
Portengen et al. [151]/The Netherlands 2005	162 pig farmers/case- control study	Not done	Modelled airborne endotoxin	Not done	Not done	+ SPT: endotoxin exp. < 75 ng m^−3^ 0.03 (0.0–0.3) endotoxin exp. > 75 ng m^−3^ 1.2 (0.4–3.6)	Endotoxin exposure: 0.9 (0.3–2.3)	Endotoxin exposure: 1.2 (0.5–2.3)	Endotoxin exposure associated with BHR in sensitized pig farmers: 17 (1.3–227)
Radon et al. [103]/Germany 2006	2678 rural adults, age 18–44 years/cross-sectional	Only in childhood: 877+ 1118−	Childhood and adulthood: 421+ 876− Only in adulthood: 75+ vs 1043−	Not done	Allergic rhinitis and farm animal exposure: only in childhood 0.7 (0.5–0.9) In childhood and adulthood 0.2 (0.1–0.4) Only in adulthood 1.0 (0.4–2.6)	Not done	+ specific IgE and farm animal exp: only in childhood 0.7 (0.5–0.9) in childhood and adulthood 0.4 (0.3–0.6) Only in adulthood 2.4 (1.1–5.2)	Not done	Adult protection = effect of self-selection?
Douwes et al. [89]/New Zealand 2007	4262 farmers vs 1314 non-farmers/cross-sectional	3081+ vs 2495−	Not done	Current and childhood exp.: asthma ever 0.6 (0.5–0.7) Wheeze 0.6 (0.5–0.7) Current exp. only: asthma ever 0.7 (0.6–0.8) wheeze 0.8 (0.6–0.99) Childhood exp. only: asthma ever 0.9 (0.6–1.2) wheeze 1.01 (0.7–1.3)	Current farming exp.: self-reported nasal sx 0.97 (0.8–1.1) Childhood exp. only: self-reported nasal sx 0.8 (0.7–0.9)	Not done	Not done	Not done	/
Chen et al. [91]/Canada 2007	579 farmers/cross-sectional study in the general population	Not done	Grain or livestock farming (85% both)	Dd asthma OR = 0.8 (0.5–1.1)	Self-reported nasal sx OR = 0.95 (0.8–1.2)	hdm, grass pollen, cat, *Alternaria* 0.7 (0.6–0.9)	Not done	Not done	/
Schulze et al. [152]/Germany 2007	1595, age 18–44 years/cross-sectional	677+ vs 918−	Not done		Farmers: dd 0.7 (0.4–1.1)	Farmers: allergic rhinitis, 0.5 (0.4–0.8)	Farmers: + SPT 0.7 (0.6–0.9)	Not done	Dd asthma in sensitized farmers: 0.5 (0.3–1.0) BHR in sensitized farmers: 0.8 (0.5–1.1)
Smit et al. [100]/The Netherlands *2007*	593 organic farmers vs 1205 conventional farmers, mean age 44–45/cross-sectional	1370+ vs 428−	911 livestock only 629 crops only 258 livestock and crops	Livestock farmers 1.0 (0.5–2.2) Livestock farmers with childhood farm exp.: 0.6 (0.4–1.2)	Livestock farmers 0,5 (0.3–0.9) Livestock farmers with childhood farm exp.: 0.4 (0.3–0.7)	Not done	Not done	Not done	No clear effect organic farming
Smit et al. [10]/The Netherlands 2008	877 farmers and agri-industry workers, mean age 40–46/cross-sectional	511+ vs 366−	Endotoxin exposure (modelled)	Farm childhood: dd 0.9 (0.3–2.8) No farm childhood: dd 0.9 (0.4–2.3) Endotoxin exp.: wheezing 1.4 (1.2–1.7) dd 0.99 (0.5–2.0)	Farm childhood: self-reported 0.6 (0.4–0.9) No farm childhood: self-reported 0.6 (0.4–0.8) Endotoxin exp.: self-reported 0.6 (0.5–0.8)				No effect modification by farm childhood
Eriksson et al. [153]/Sweden 2010	18,087 rural population/cross-sectional	2557+ vs 15,238–	Urbanization	Not done	Raised on a farm: self-reported 0.8 (0.7–0.9)	Not done	Not done	Not done	/
Smit et al. [101]/The Netherlands 2010	427 farmers	193+ vs 234−	Endotoxin exposure (modelled)	Endotoxin exposure: wheezing 1.3 (1.01–1.7)	Endotoxin exposure: self-reported 0.6 (0.4–0.7)	Not done	Endotoxin exp.: specific IgE to common allergens 0.7 (0.5–0.8)	Endotoxin exposure: total IgE 0.9 (0.7–1.05)	Effects on sensitization mainly in non-FC Endotoxin exposure: BHR 1.5 (1.03–2.3)
Basinas et al. [7]/Denmark and The Netherlands 2012	3883 farmers, veterinary students and power plants workers/cross-sectional	+ (adjusted)	JEM-estimated airborne endotoxin: four levels; reference ≤ 50 EU m^−3^	High vs low occup. endotoxin exposure: wheezing 1.7 (1.1–2.6) asthma 1.5 (1.1–2.1)	High vs low occupational endotoxin exposure: hay fever 0.6 (0.4–0.9)	High vs low occup. endotoxin exposure: positive SPT and/or IgE to pollen, hdm, and pets 0.7 (0.4–0.99)		Not done	/
Galli et al. [93]/Italy 2015	78 Italian swine farmers vs 82 non-swine farmers/cross-sectional	Not stated	Swine farming vs non-swine farmers	6.4% vs 15.8%, p < 0.06	16.7% vs 51.2%, p < 0.01	+ SPT to grass: 7.7% vs 25.6%, p < 0.02	Not done	Not done	/
Rennie et al. [154]/Canada 2015	1599 rural adults	1068+ vs 531–	766+ vs 833−	Not done	Not done	Women living on a farm in the 1st yr. of life: atopy (positive SPT) 0.6 (0.4–0.9)	Not done	Not done	/

*dd* doctor-diagnosed, *BHR* bronchial hyperresponsiveness, *sx* symptoms, *hdm* house dust mite, *SPT* skin prick tests, *JEM* job exposure matrix, *Lep d* Lepidoglyphus destructor

The widespread knowledge of the farm-associated low risk of atopy may easily lead to a common but incorrect belief that “the farm environment protects against asthma and rhinitis”. As emphasized in this position paper, farm work remains a major risk factor for (mostly non-atopic) LRT and URT illness and the ‘anti-atopy’ effect is mainly a complicating factor in the diagnostic workup. A clear distinction between atopic and non-atopic respiratory disease is thus essential. Studies in both adults and children have found that high endotoxin exposure, although negatively associated with atopic asthma—defined as wheezing illness combined with atopic sensitization -, is positively associated with wheezing in the absence of atopy [89]. The meta-analysis of studies with objectively determined atopy markers—SPT or IgE positivity—found as most consistent finding protection by both a farm childhood and adult farm work against atopic sensitization, especially against pollen [42]. Most population studies however did not clearly distinguish between atopic sensitization and associated illness. Hence, the often-reported protection against “(atopic) asthma” by a farm childhood may primarily reflect protection against atopy, and less against wheezing illness as such. In the farm work environment, with its much higher airborne microbial exposures, the risk of non-atopic wheezing may prevail, so that beneficial effects preventing atopy are outweighed by the enhanced risk of innate immunity-mediated non-allergic (non-atopic) respiratory disease.

### Exposure and prevention

In farming occupations there is a challenge for exposure assessment, due to the many different substances, see Table 4. Details related to the methods available for monitoring dust, microbial and allergen concentrations in occupational as well as environmental settings have been published elsewhere [105–110]. For a detailed review on other exposures in farming, please see [1, 110–112].

**Table 4:** Bioaerosol-components in farming environment

Substance	Method of determination
Allergens	Antibody-based assays (sandwich) ELISA
Bacteria and Vira	Viable sampling, microscopic analysis of samples, Non culture-based microbiological markers or surrogate markers such as endotoxin (Gram negatives), muramic acid (Gram positives) DNA or RNA based molecular methods ranging from qPCR to 16S microbiome or full metagenomic analysis C
Endotoxin	Classical “LAL-test” (kinetic chromogenic test) or recombinant factor C assay
Beta(1 → 3) glucan	Factor G pathway of the LAL-test or poly-/monoclonal antibody assays (ELISA)
Pyrogenic activity	Whole blood assay (outcome: IL-1β, IL-6 release)
Moulds	Cultivation of fungi Non culture-based microbiological marker Surrogate markers like ergosterol or extra-cellular polysaccharides specific for Pen/Asp (EPS) DNA or RNA based molecular methods ranging from qPCR to ITS or full metagenomic analysis
Fungal fragments	Non-gonomorphic particles (Halogen immunoassay)
Mycotoxins	ELISA LC–MS (indirect assessment by analyzing settle dust) Biomonitoring

#### Exposure levels

Evidently, most of the available data on workplace exposure levels concern dust, endotoxins and (1 → 3)-β-d-glucans. Organic dust is frequently used as a marker of exposure to bio-aerosols whereas information regarding levels of other airborne exposures is scarce. Readers interested in such studies are recommended to look elsewhere [37, 113].

Overall, studies have shown great variations in personal exposures both between and within different farm types (Table 5). Average personal concentrations of dust are reported to range between 0.2 and 11.2 mg m^−3^ with content of endotoxin and glucan concentrations averaging between 13 and 9609 EU m^−3^ and 223 and 10,300 ng m^−3^, respectively. Pig and poultry farmers are the highest exposed, whereas mixed production and mink-farmers are the lowest exposed, irrespectively of the agent concerned. The available data related to airborne levels of specific allergens in stables are limited, however, to dairy and horse stables. Samadi et al. measured personal and stationary levels of bovine (*Bos d 2*) allergens in 23 diary stables in the Netherlands [114]. Personal levels of exposure ranged from 0.10 to 46.8 μg/m^−3^ with an average (GM) of 1.47 µg m^−3^, and were generally higher than the measured stationary levels (GM = 0.66 μg m^−3^; range: 0.03 to 35.6 µg m^−3^). These concentrations generally exceed those reported in the only earlier study available concerning levels among Finish diary barns by 2 to 3 folds [115]. Similar deviations have been reported in average allergen concentrations measured within horse stables [116–118].

**Table 5:** Overview of results from studies of airborne dust, endotoxin, (1 → 3)-β-d-glucan and allergen levels within farm workplaces. Personal exposure levels from the inhalable and/or total fraction are summarized except when indicated

Environment	Dust (EU m^−3^)		Endotoxin (EU m^−3^)			(1 → 3)-β-d-glucan (ng m^−3^)			Allergens (U m^−3^)			
	Range of means (individual concentrations)	References	Range of means (individual concentrations)	Analytical method	References	Range of means (individual concentrations)	Analytical method	References	Agent	Range of means (individual concentrations)	Analytical method	References
Livestock farming												
Pig farming	0.83–5 (< LOD–76.7)	[10, 126, 129, 155–157]	400–3400 (< LOD–374,000)	KC/T-LAL, rFC	[10, 126, 129, 156, 157]	223 (6–5208)	Glucatell (Factor G LAL)	[157]				
						NR (33–410)	SI-EIA	[158]				
						NR (18–96)	Glucatell (Factor G LAL)	[158]				
Dairy farming	0.6–2.4 (< LOD-9.8)	[10, 119, 129, 130, 135, 159–162]	220–1570 (< LOD–8290)	KC/T-LAL, rFC	[10, 119, 129, 130, 135, 159–162]	10,300 (150–232,000)	SI-EIA	[135]	Bovine allergen	1.39 (0.1–46.8)	ELISA	[114]
Poultry farming, non-specific	6.5–7.0 (0.02–81.3)	[156, 163]	2576 (190–16,348)	KC/T-LAL	[156]	NR (13–5000)	Glucatell (Factor G LAL)	[158]				
						NR (2–972)	SI-EIA	[158]				
Poultry farming, layers	2.4–9.6 (1.6–14)	[129, 162, 164, 165]	694–7517 (1162–19,745)	KC/T-LAL, rFC	[129, 162, 164, 165]							
Poultry farming, broilers	2.2–11.2 (4–4.4)	[162, 164]	596–9609 (61–8120)	KC/T-LAL	[162, 164]							
Mink farming	1.3 (0.5–2.3)	[129]	214 (93–1050)	KC/T-LAL	[129]							
Mixed livestock production farming	0.54–1.9 (0.4–8.9)	[129, 160]	448 (< LOD-2910)	KC/T-LAL	[129]							
Horse keeping/farming	1.4 (0.2–9.5)	[116]	742 (92–9846)	KC/T-LAL	[116]	9500 (< LOD–631,000)	SI-EIA	[116]	Horse allergen	ELISA	438–4300 (286–6272)*#	[117, 118]

*NR* not reported, *LOD* limit of detection, *LAL* limulus amebocyte lysate (LAL) assay, *KC/T-LAL* kinetic and/or turbidimetric chromogenic LAL assay, *rFC* recombinant factor C assay, *SI-EIA* specific inhibition enzyme-linked immuno assay, *Glucatell* glucatell modification of the LAL assay, *ELISA* enzyme-linked immunosorbent assay

* Transformed from U mg^−3^ assuming 1 U = 1 ng

^#^Stationary measurements

Other important biological agents include ergosterol, muramic acid [119] and mycotoxins [120–122]. Ergosterol and muramic acid are considered markers for exposures to fungal and Gram-negative bacterial, respectively. The health effects of mycotoxins are well described, but their quantification within workplace environments, including farming, remains poor [113].

Exposure studies employing repeated measurements (i.e. measuring the same workers on more than one working day) suggest that the levels of exposure to bio-aerosols vary considerably both across different days for the same worker and between different workers that perform the same job [1, 114, 123]. A recent systematic review suggested that average levels of personal dust and endotoxin exposures in livestock farming remained relatively unchanged (i.e. no temporal trends were observed) in the period between 1985 and 2013 [1]. In a more elaborated approach an almost 2% annual decline in exposure was revealed for the period 1992–2008. The utilized exposure database did not solely comprise measurements from primary agriculture production, and when models were restricted to measurements only from pig farming no time trends seemed to be present (Basinas et al. in preparation).

#### Factors affecting exposure during farm work

Bio-aerosol sources are abundant in both indoor and outdoor farm working environments. The environmental conditions and workplace characteristics, as well as the activities performed, are suggested to determine the personal exposures of farmers. Previous research has shown that personal exposures are highest during stable activities involving feed handling, distribution of bedding, intense handling of active animals (e.g. weighing, transport, re-penning and loading) and high pressure washing [43, 111, 124–128] and lowest during field work, and for cattle farming, the repair of stables and the hosing of parlours following end of the milking process [128–130]. Grain threshing and handling related activities such as storage have also been reported to increase personal levels of bio-aerosol exposures [131].

Besides working tasks, the effect of environmental and farm characteristics has also been assessed in a few studies, of which some have been performed in years prior to the ones covered by the present review (Table 6). Feeding, flooring and ventilation parameters (e.g. type, coverage, system employed) have also been suggested to be strong predictors of in-door personal exposure levels to bio-aerosols [43, 111, 124, 132, 133]. An increased outdoor temperature and the summer season, both indicators of high ventilation rates, have been shown to decrease personal levels of exposure for workers in stables irrespectively of the type of production involved [43, 111, 119, 124, 126, 128, 129, 133, 134]. The general hygiene within the stable has also been shown to influence exposure, whereas for poultry farmers factors such as the age of the chickens involved and the housing system (e.g. aviary vs cage) seem to be of importance. An interesting and consistent observation in recent studies, is a strong association of robot milking in diary stables with an increased exposure of workers to dust and glucans [114, 128, 135]. This effect has been suggested to reflect altered working patterns combined with an increased ratio of animals per worker [128]. Such results of process alterations may be apparent also in other types of production influenced by the tendency towards enlarged productions in Western countries resulting in workers that have less intermittent working tasks and thus more permanent patterns of exposure [1]. Hence, there is an increased demand for effective exposure control and prevention strategies for such workers.

**Table 6:** Literature reported engineering and production parameters affecting personal exposures of farmers to bio-aerosols

Determinant	Substance	Factor	Estimated effect	Source
Pigs				
Environment	Dust, endotoxin	Season, summer	Lower levels of exposure compared to winter	[43, 124, 126, 129]
	Dust, endotoxin	Outdoor temperature	18–36% decrease in levels per 10 °C increase in temperature	[43, 124]
Production stage	Dust	Finishing units	Exposures highest in finishing and/or weaning stables and lowest in farrowing and/or breading.	[166, 167]
Ventilation	Dust	Negative pressure	lower exposures compared with neutral or mixed methods by 26–50%	[43]
	Dust, endotoxin	Air exhaust via other compartments or the pit	Increased exposures relative to when characteristic not present by 28–42%	[124]
	Endotoxin	Use of a showering system	7% increase of exposure per 10 min spent on presence of characteristic	[43]
Feeding	Dust	Automatic feeding	Lower exposures with increased time spent on presence	[124]
	Dust, endotoxin	Wet feed	Lower levels when compared with dry feed by 21–79%	[43, 124]
	Dust	Fat in feed	Increased fat content associated with lower levels of exposure	[132]
	Dust	Ad libitum feeding	5% increase in levels per 10 min spent on presence of the characteristic	[43]
Flooring	Endotoxin	Full slatted floor	Full slatted floor associated with increased exposure levels by 50% compared with a full concrete or 16% for every 10 min spent on presence	[43, 124]
	Dust	Fully concrete floor	Fully concrete floor associated with 21% decrease in dust exposure	[124]
	Endotoxin	floor heating	38% increase in exposures per 10 min spent on presence	[124]
General hygiene	Dust, endotoxin	Very dusty stable	7–18% increased exposure compared to a non-dusty environment	[124]
	Dust	Wet floor	Reduced levels compared to dry floor by 12%	[168]
Other	Dust	Ventilation and floor, and manure type combinations	Exposures lowest in natural ventilated buildings with slatted floors. Highest exposures in mechanically ventilated buildings with scrapper manure collection.	[169]
Cattle				
Environment	Endotoxin	Outdoor temperature	≥ 18% decrease in levels per 10oC increase in temperature	[111, 119, 128]
Feeding	Endotoxin	Semi-automatic system	42% reduction compared to manual feeding	[111]
	Dust	Amount of feed (pellet, meal)	2% increase in exposure per kg distributed	[111]
Bedding	Dust, endotoxin, glucans	Compost bedding	Compost bedding associated with higher exposures compared to rubber mats by 5% for dust and 179 to 400% for the constituents	[114, 135]
Animal density	Dust, endotoxin, bovine allergens	Surface area per cow	Increased surface associated with decreased levels of exposure by 7 to 65%	[114, 115, 135]
Manure handling	Dust	Automatic scrapers in alley ways	40% reduction compared to when system not used	[128]
	Endotoxin	Slope or back flashed system in pit	175% increase compared to round or scraper based systems	[128]
Milking	Dust, glucans, bovine allergens	Robot	Robots associated to increased exposure compared to parlour milking by 22–86% for dust and 138% for glucans but decreased exposures to bovine allergens by 65%.	[114, 128, 135]
General hygiene	Dust, endotoxin	Parlour cleaning	Increased frequency of parlor cleaning associated with lower levels of dust and endotoxin	[170]
Poultry				
Environment	Dust, endotoxin	Season, summer	Somewhat lower levels of exposure compared to winter for layers, and turkey farmers	[133, 134]
Barn system	Dust, endotoxin	Floor (aviary)	Floor (Aviary) housing system results in higher concentrations relatively to cage housing	[165, 171, 172]
	Dust	Enclosed system	Higher exposures in systems that are enclosed (only mechanical ventilated) compared to those being open with both mechanical and natural ventilation present	[134]
Production stage	Dust, endotoxin	Flock age	Increased flock age associated with decreased exposures	[129, 134, 164]
	Dust, endotoxin	Parent stock	Levels in parent stock farm higher compared to broiler and layers	[134]
	Dust, endotoxin	Hen (Turkey)	Levels in hen stables higher compared to those of toms and brooders	[133]
Ventilation	Dust, endotoxin	Ventilation rate	Increased ventilation rate related to decreased levels of exposure	[133]
General hygiene	Dust, endotoxin	Litter presence in control alleys	Presence of litter in control alleys assoc. with higher exposures compared to no presence	[134]
Other	Dust, endotoxin	Tilling of litter	Performance of litter tilling related with increased levels of exposure	[133]

### Preventive interventions in farming workers

Although the farm environment is considered to be allergenic, irritant and toxic for human airways, farmers’ knowledge about occupational risks and safety rules seems to be modest [68, 136] and medical recognitions of farm WR respiratory diseases are underestimated [137]. The results of 14-year study including nearly 3500 farmers with occupational diseases indicate the necessity for implementing periodic health examination programs and improving working conditions of agricultural workers [138]. One study of exposure levels was able to demonstrate an effect of feed-back vs no feed-back to the farmers on their own exposure level plus the mean of the other farms. In this study feed-back was associated with lower levels during a repeated measuring campaign 6 months later [139]. Programs based solely on increased use of respirators may not be effective and/or efficient in depth of time; respirator use is as a low tier prevention approach with efficiency strongly dependent on type, proper use and worker behavior [140]. In asthma and rhinitis, avoidance of further exposure to causal agents is recommended, but this may not be achievable in farming populations, mainly due to socio-economic considerations. Therefore a comprehensive strategy of combining interventions towards reduction of harmful workplace exposures, with periodic medical check-ups and treatment optimization is urgently needed.

### Research needs

In each of the preceding chapters, serious gaps in current knowledge of rhinitis and asthma in livestock farmers are identified that require well-designed future research.

- Follow-up studies: Most population studies had primarily a cross-section design, and only a few also a longitudinal follow-up over periods of more than 2–5 years. Most worthwhile would be studies in which the long-term development of respiratory health (symptom prevalence and severity, BHR, lung function, allergic sensitization) is monitored in farmers with and without more or less severe symptoms, and who either left farming, or remained in farm work with or without changing work practices or jobs within agriculture such that exposures were strongly diminished.
- Mechanisms and diagnosis: The pathophysiology of respiratory disease in farmers has been thoroughly studied, including the role of various cell types, cytokines, etc., in innate immunity reactions that may be the predominating cause of most farm and microbial dust-induced illness. In contrast to type I allergy, where specific SPTs or IgE tests and measurement of occupational allergens can be used. Hence, there are no diagnostic tools available with which clinicians can identify innate immunity-mediated reactions to farm and microbial dust causing URT and LRT illness in farmers. Future research thus may focus on development of tests of markers of acute or chronic innate immunity reactions (e.g. patterns of cytokines in blood, nasal or bronchial lavages). Such tests should—possibly in combination with other markers like BHR, and with the help of more sophisticated algorithms—improve diagnosis and prognosis of farm dust and livestock-associated respiratory disease.
- Prevention and intervention: intervention measures have been largely limited to educational activities and incidental studies on effectiveness of technical measures to reduce dust and microbial exposures and use of personal protective devices. Further studies need to include more systematic studies with sufficient power and follow-up to assess effects of interventions both on exposure levels and on the respiratory health of participants.

## Conclusion

In spite of technological changes, the over-all levels of airborne exposure of livestock farmers to organic dusts, including microbial agents and allergens, ammonia and other gases, haven’t changed considerably and remained high and is still a serious health hazard.

Accordingly, prevalence and incidence of work-related respiratory disease, including asthma, bronchitis and upper respiratory tract symptoms among workers in livestock farming have remained high.

Causal factors and mechanisms may in some cases be specific farm allergens and IgE-mediated type I sensitization—to e.g. storage mite, bovine or horse allergens –, but the large majority of work-related respiratory symptoms in livestock farmers is caused by innate immunity responses to microbial agents like bacterial endotoxins, glucans and other innate immunity stimulating agents, thus leading to ‘non-allergic asthma’ and bronchitis.

A thorough anamnesis and identification of symptoms as clearly exposure-associated is the key point in the diagnosis of work related upper- and lower respiratory tract diseases in farmers. Even if common atopy and NSBHR are strong risk factors, the diagnostic procedure cannot depend entirely on IgE serology, specific inhalation challenge or other tests for specific immunologic sensitization.

Since many farm workers have been raised on a farm, the well-known protective effect of a farm childhood against atopic sensitization, allergic asthma and rhinitis can also be found in adult farm workers. Results of several studies suggest that farm exposure in adulthood may provide an additional protective effect. This protection however appears to be largely limited to atopic sensitization, particularly to pollen, and hardly affects the enhanced risk of non-allergic asthma in farm workers.

## Supplementary information

**Additional file 1.** Appendix S1 Search strategy.

## Data Availability

Not applicable.

## Abbreviations

BHR: Bronchial hyper-responsiveness
CAFOs: Concentrated animal feeding operation
ECRHS: European Community Respiratory Health Survey
HWS: Healthy worker selection
LRT: Lower respiratory tract
NOD: Nucleotide-binding oligomerization domain
NSBHR: Nonspecific bronchial hyper-responsiveness
ODTS: Organic dust toxic syndrome
PAMPs/MAMPs: Pathogen- and microbial-associated molecular patterns
PGRPs: Peptidoglycan recognition proteins
SIC: Specific inhalation challenge
SPTs: Skin prick tests
SUS: Study of young farmers
TLR: Toll-like receptors
URT: Upper respiratory tract
LRT: Lower respiratory tract
WR: Work-related
