# Supplementary material for: Respiratory diseases and allergy in farmers working with livestock: a EAACI position paper
Source: Clin Transl Allergy. 2020 Jul 6;10:29. doi: 10.1186/s13601-020-00334-x (PMC7336421; doi:10.1186/s13601-020-00334-x)
Supplement: Supplementary file 1 — Additional file 1. Appendix S1 Search strategy. [file 13601_2020_334_MOESM1_ESM.pdf]

Respiratory diseases and allergy in farmers working with large animals and Poultry: A EAACI Position paper

Appendix 1 Search strategy *Update: (sept 2019)*

1.1 Restrictions:

1. Farm work-related exposures
2. Livestock: cattle or/or swine farming
3. (work-related) asthma and rhinitis and COPD, in adulthood

1.2 Exclusion

- farm childhood exposure *vs* asthma/rhinitis/allergy in children
- ODTS, HP
- URT and LRT infectious disease

1.3 Comments/questions & in/exclusion:

Focus *(see also objectives of Task Force)***:**

1. Epidemiological studies on incidence/prevalence of asthma and rhinitis in farmers/farm workers
2. Studies on exposure in livestock farming – any (major) qualitative (other agents?) or quantitative changes during last decades, due to upscaling of livestock farming?
3. Dose-response relations between exposure levels and the risk of asthma/rhinitis among farmers/farm worker?
4. new insights re the pathogenesis of farm-related asthma and rhinitis
5. The paradox: farm exposure's *protection* against asthma/allergy/rhinitis ←→ farm-exposure as *risk factor* for asthma and rhinitis?
6. Prevention measures: effectiveness of primary and/or secondary prevention?

For these topics strict focus on ***primary literature*** *from the* ***last 10-15 years*** *– studies performed (=data collected) after 2000.* In case of abundance in one or more of the 5 focus areas, selection with as criteria:

- ~equal numbers of primary papers (if possible, at least 5-10) in each of the six focus areas;
- date of publication (or better: study date/period); priority for most recent papers
- geographical distribution/coverage – trying to incorporate studies from a variety of geographic regions worldwide (restriction to industrialized world)

Literature Search strategy (MEDLINE/PubMed)

1.3.1 Step A:

A series of papers on respiratory health risks (priority: asthma and rhinitis) and associated exposure factors in livestock farming; published 2011-17 – mainly reviews:

Authors	*Journal*	*PY*	*Topic*
**Basinas et al.**	J Exp Sci Environ Epidemiol	2015	Exposure
**Bolund**	Occup Environ Med	2017	Dose-response lung function
**Luong Thanh**	Cochrane Rev	2016	Prevention in Occup settings
**May**	J Tox Env Health	2012	Mechanisms
**Nordgren & Bailey**	Curr Op Resp Med	2016	General/clinical/diagnosis
**Poole**	Ann Asthma Allergy Immunol	2012	Exposure factors
**Poole & Romberger**	Curr Op Allergy Clin Immunol	2012	Mechanisms
**Reynolds**	J Agromed	2013	General dairy farming
**von Essen**	J Agromed	2010	Prevention/NIOSH recommendations
**Wunschel & Poole**	J Asthma	2016	General/risk factors

1.3.2 Step B:

Inventory of all MeSH terms added in MEDLINE to these papers; MESH terms sorted on frequency with which they were. After exclusion of terms considered non-specific or not relevant, and terms used only once (numbers give frequency of use):

MeSH term	#
Occupational Exposure	12
Animals	8
Endotoxins/adverse effects	7
Dust	7
Lung Diseases/chemically induced*	6
Air Pollutants, Occupational/adverse effects	5
Occupational Diseases*/epidemiology	5
Pulmonary Disease, Chronic Obstructive/epidemiology	5
Agriculture*	4
Agricultural Workers' Diseases/chemically induced*	4
Asthma/epidemiology	4
Animal Husbandry*	3
Respiratory Tract Diseases/epidemiology	3
Bronchitis, Chronic/epidemiology	3
Aerosols	2
Cattle	2
Dairying	2
Livestock	2
Swine	2
Hypersensitivity/epidemiology	2

1.4.3 Step C:

Check position of these terms in MeSH thesaurus tree (eg. asthma and rhinitis in the tree of ‘respiratory tract diseases’; ‘livestock’ in tree ‘animals’)

1.4.4 Step D:

Search in Pubmed/MEDLINE for items indexed with “MESH terms” or “MESH major topics”

Search in MEDLINE for items indexed with MESH terms or MESH major topics				
Set	*Search*	*Search term / combination*		#hits
1	#2	Occupational exposure	MeSH	61.209
2	#3	Occupational exposure/adverse effects	MeSH	19.523
3	#4	Dust	MeSH	21.590
4	#5	Aerosols	MeSH	30.333
5	#6	Endotoxins/adverse effects	MeSH	13.780
6	#7	Air pollutants, Occupational/adverse effects	MeSH	7.081
A		*ANY (#2;#4-#7)*		*124.447*
A1		*ANY (#3;#4-#7)*		*86.933*
7	#12	Agriculture	MeSH	91.051
8	#13	Animal husbandry	MeSH	20.401
9	#14	Dairying	MeSH	11.729
10	#15	Farms	MeSH	1.631
11	#16	Cattle	MeSH	341.390
12	#20	Swine	MeSH	231.576
13	#21	Livestock	MeSH	3.184
B		*ANY (#12- #16; #20-#21)*		*608.084*
14	#23	Respiratory tract diseases	MeSH	1.295.916
15	#24	Asthma	MeSH	124.190
16	#25	Rhinitis	MeSH	33.156
17	#26	Pulmonary Disease, Chronic Obstructive	MeSH	52.575
18	#27	Lung diseases	MeSH	851.713
19	#28	Bronchitis, chronic	MeSH	1.724
20	#29	Hypersensitivity	MeSH	334.692
C		*ANY (#4; #5; #12; #25; #27; #28; #29)*		*1.496.523*
21	#31	Agricultural Workers' Diseases	MeSH	6.535
22	#32	Farmers OR workers OR occupational	Title or Abstract	261.287
D		*#31OR #32*		*264.470*
E		*A1 AND B AND C*		*798*
F		*E and D*		***377***
G		F and PY >1999 (published since 2000)		***266***
